# Performance Study of Piezoelectric Injection System Based on Finite Element Simulation

**DOI:** 10.3390/mi14040738

**Published:** 2023-03-26

**Authors:** Xin Li, Yongsheng Zhao

**Affiliations:** Department of Materials and Manufacturing, Beijing University of Technology, Beijing 100124, China

**Keywords:** additive manufacturing, piezoelectric injection system, FES, jetting velocity, droplet diameter

## Abstract

This paper presents a performance prediction method for piezoelectric injection systems, based on finite element simulations. Two indexes representing the system performance are proposed: jetting velocity and droplet diameter. By combining Taguchi’s orthogonal array method and finite element simulation (FES), a finite element model of the droplet injection process, with different parameter combinations, was established. The two performance indexes, jetting velocity and droplet diameter, were accurately predicted, and their variation with time were investigated. Finally, the accuracy of the predicted results of the FES model was verified by experiments. The errors of the predicted jetting velocity and droplet diameter were 3.02% and 2.20%, respectively. It is verified that the proposed method has better reliability and robustness than the traditional method.

## 1. Introduction

Additive manufacturing (AM) enables the design of parts that cannot be easily fabricated with traditional manufacturing techniques, and offers great opportunities for a wide range of applications in industries including aerospace, automotive, defense, and biomedical [1]. Additive manufacturing, also known as 3D printing, is divided into two methods: drop-on-demand (DOD) and continuous drop [2]. Since the DOD method produces individual droplets through rapid changes in the volume of the cavity [3], it can reduce material waste, while having high print quality [4]. Non-contact piezoelectric injection, a DOD mode droplet injection process, is characterized by its fast response time and the ability to change the diameter and injection speed of droplets in real time, by adjusting the frequency [5]. Achieving droplet injection, accuracy and uniformity of piezoelectric injection is of great importance, because of the increasing quality requirements for droplet injection in manufacturing processes [6].

Despite the many advantages of piezoelectric jetting, problems such as over-jetting or discontinuities may arise, due to a variety of factors such as material properties, substrate characteristics, and process parameters [7]. Among the many factors affecting the quality of jet printing, process parameters (jetting velocity, jet height, and substrate morphology, etc.) are preferred for optimizing the non-contact piezoelectric jetting process. Among them, the injection speed affects not only the volume and diameter of the droplet, but also the shape and uniformity of the droplet when they are injected onto the substrate surface. In the piezoelectric injection system, the dimensional parameters of the nozzle and needle are the key to the droplet jetting velocity; the larger the nozzle diameter, the faster the jetting velocity. All other things being equal, the greater the nozzle angle, the slower the jetting velocity. [8]. Many studies still optimize and design piezoelectric injection system parameters through experience.

Some researchers have conducted experiments on the parametric design of nozzles and needles. Based on the basic principles of fluid dynamics, Bartolo et al. [9] used computational fluid dynamics modeling to measure material properties (viscosity); Mao et al. [10] proposed a soft fluidic roller, using a simple structure composed of a bendable and twistable electrohydrodynamic (EHD) pump and a layer of natural latex; K.S. Kwon et al. [11] measured process parameters (contact angle) using fluid dynamics modeling; Mao et al. [12] studied a bendable and twistable electrohydraulic pump, by means of digital fabrication; and M. Tsai et al. [13] investigated the effect of pulsed voltage on droplet injection behavior.

In this paper, two metrics are proposed to describe the performance of piezoelectric injection systems, namely the jetting velocity and the droplet diameter. Finite element models, with different combinations of parameters, are designed using the orthogonal experimental method. The variation law of the performance indexes with time is predicted by the established finite element models. Finally, the predicted results of the finite element simulations are compared with the experimental results, to verify the validity and accuracy of the proposed model.

## 2. Finite Element Modeling and Simulation of Droplet Ejection Process

### 2.1. The Process of Building the Finite Element Model

#### 2.1.1. CAD Model

**Figure 1:**
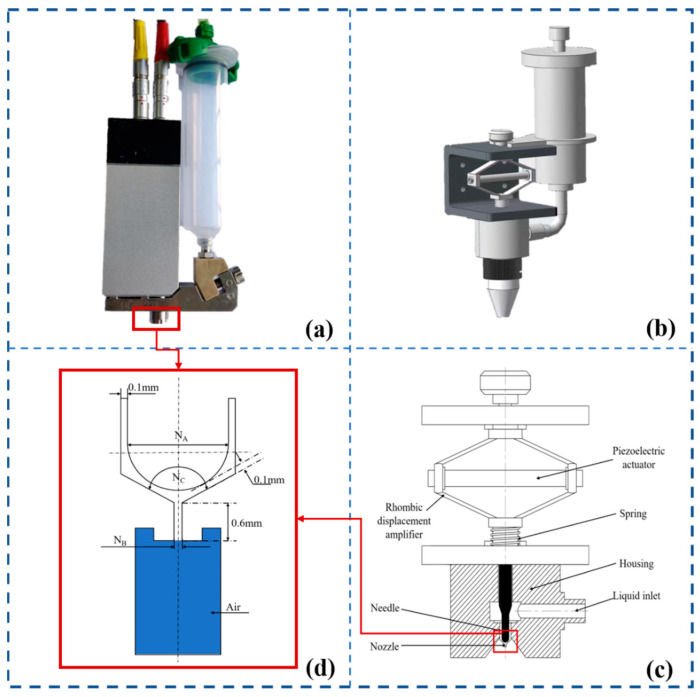
Design of the studied piezoelectric ejection system and dimensions of core components (nozzle and needle). (**a**) Physical drawings, (**b**) three-dimensional drawings, (**c**) two-dimensional simplified drawings, (**d**) two-dimensional drawings of core components.

The physical, three-dimensional and two-dimensional simplified drawings of the model of the piezoelectric-driven droplet injection system designed in this paper, are shown as (a), (b), and (c) in Figure 1, respectively. Where (d) is the structural diagram of the core components of the system (nozzle and needle). In this paper, the nozzle design parameters $N_{A}$, $N_{B}$, $N_{C}$, and $N_{D}$ are selected as the parameters to study the design of the droplet injection system, representing the needle diameter, nozzle diameter, nozzle angle, and piezoelectric-driven needle motion speed parameters, respectively. 

#### 2.1.2. Theoretical Model

In this paper, a multi-coupled phase change model of fluid flow was developed, using the ANSYS Fluent software. The software uses the VOF algorithm to simulate and analyze the fluid flow [14]. In the VOF algorithm, the incompatible fluid components share a common set of momentum equations, and the tracking of the interphase interface in the computational domain is achieved by introducing a variable Q, which represents the phase volume fraction [15].

(1) $$Q=\left\{ \begin{array}{l} 0\;\;\;\;\left(\mathrm{outside}\;\mathrm{the}\;\mathrm{liquid}\right)\\ 0<Q<1\;\;\;\;\left(\mathrm{on}\;\mathrm{the}\;\mathrm{interface}\right)\\ 1\;\;\;\;\left(\mathrm{inside}\;\mathrm{the}\;\mathrm{liquid}\right) \end{array}\right.$$

Many studies [16,17,18,19] have been conducted to obtain the performance of piezoelectric injection based on Weber number, We, and Ohnesorge number, Oh.

(2) $$We=\frac{\rho \nu^{2}L}{\sigma }$$

(3) $$Oh=\frac{\mu }{\sqrt{\rho \sigma L}}$$

where ρ, μ and σ are the density, viscosity, and surface tension of the fluid, respectively, L is the characteristic length, equal to the diameter of the nozzle, and *v* is the velocity of the droplet.

The finite element simulation can be set to a computational environment close to the experimental conditions, thus ensuring that the results of the numerical calculations are closer to the experimental results. In the developed model, the following assumptions were made:

(i) the fluid is an incompressible Newtonian fluid,
(ii) the jetting time is short and the influence of temperature gradient can be ignored.

Since the heat exchange in incompressible fluids is small and negligible, the controlling equations for the droplet ejection process are established using the mass and momentum equations, without considering the law of conservation of energy, and the evolution of the flow field is simulated as follows [20]:

Continuity equation

(4) $$\frac{\partial u_{i}}{\partial x_{i}}=0$$

Navier–Stokes (momentum) equation

(5) $$-\frac{1}{\rho }\nabla p+\frac{\mu }{\rho }\nabla^{2}u+F=\frac{\partial u}{\partial t}+\left(u\cdot \nabla \right)u$$

where ρ is the density of the fluid, p is the pressure, μ is the viscosity of the fluid, u is the velocity, ∂u/∂t represents the dependent component of inertial force on the fluid, F is the mass force per unit mass fluid in momentum, and ∇p is the pressure term which exists in the form of the pressure gradient.

#### 2.1.3. Generate Meshes and Boundary Conditions

**Figure 2:**
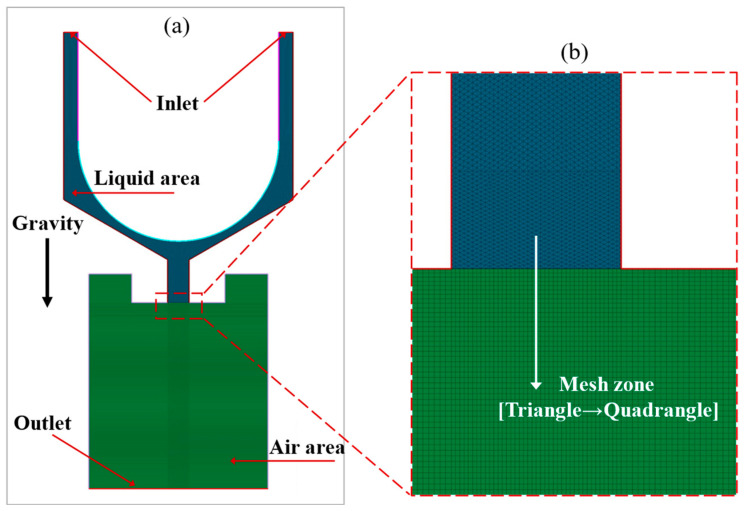
The studied model: (**a**) areas and boundaries of the FE model, and (**b**) FE mesh.

In the model developed in this study, only one half of the problem under study needs to be modeled since it is symmetric (geometry, materials, loads, and boundary conditions). As shown in Figure 2, the FE model is meshed using triangles and quadrilaterals.

The specific parameters of the fluid material used in this study, at 27 °C, are shown in Table 1. The pressure at the inlet was kept constant at 0.05 MPa, and the needle stroke was 0.1 mm, during the whole injection process, without considering heat transfer.

### 2.2. Finite Element Model

**Figure 3:**
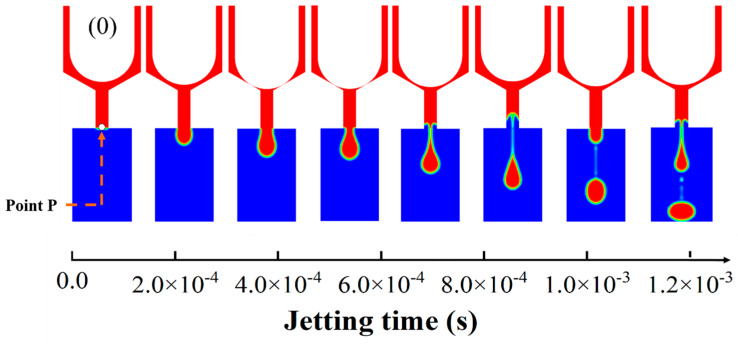
Evolution of droplets with time.

In this paper, the validity of the proposed finite element model is verified by comparing the simulation results with literature data. Second, the velocity of the P point during the simulation is compared with the experimental measurements, to verify the accuracy. Figure 3 shows the progressive evolution of the droplet shape during the finite element simulation. It agrees well with the experimental observations in the literature [21].

Due to the relatively large number of DESD parameters affecting DEB during piezoelectric injection, it is impractical to build the full combination of $N_{A}$, $N_{B}$, $N_{C}$, and $N_{D}$ (Figure 1). The sampling method can be used to draw samples from the overall population for model prediction, and cover the whole experimental area with a reasonable number of samples. It is known from the literature that there are many different sampling methods, commonly used methods include stratified sampling, Taguchi sequential sampling, and random sampling [22,23]. Since a Taguchi orthogonal array is considered useful for studying when there are interactions between variables, results can be obtained by evaluating a small number of samples [24]. Therefore, a Taguchi orthogonal array (L25) is used to create the 30 cases [25]. Table 2 summarizes all combinations of design parameters and their corresponding values of jetting velocity and droplet diameter. In this study, the same fluid material, boundary conditions, and mesh size were used in all cases, to calculate the droplet diameter and jetting velocity.

**Figure 4:**
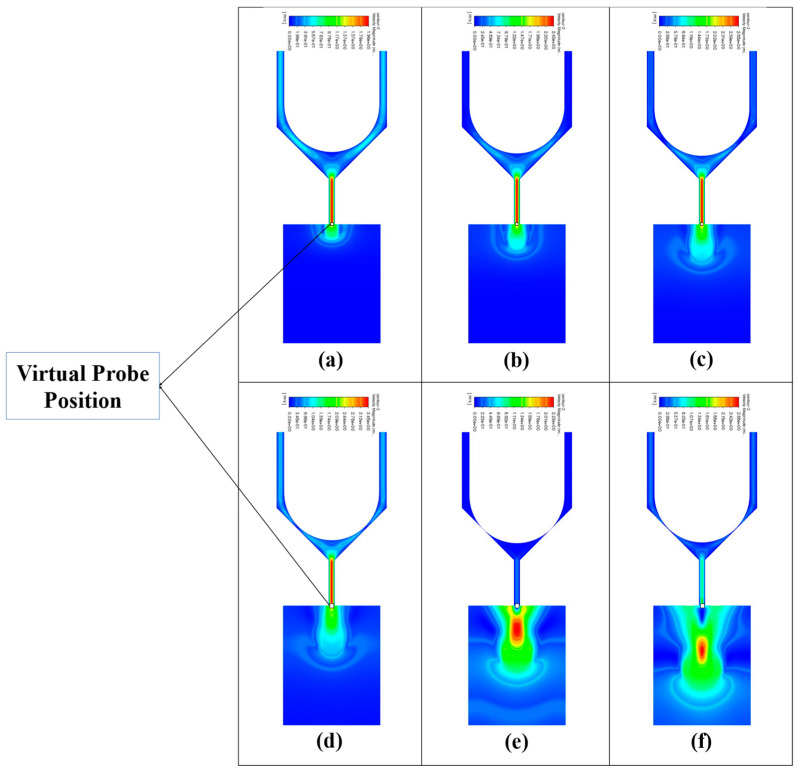
Velocity variation of the virtual probe (point P) in case 7 at different moments: (**a**) time = 0.0002 s, (**b**) time = 0.0004 s, (**c**) time = 0.0006 s, (**d**) time = 0.0008 s, (**e**) time = 0.001 s, (**f**) = 0.0012 s.

**Figure 5:**
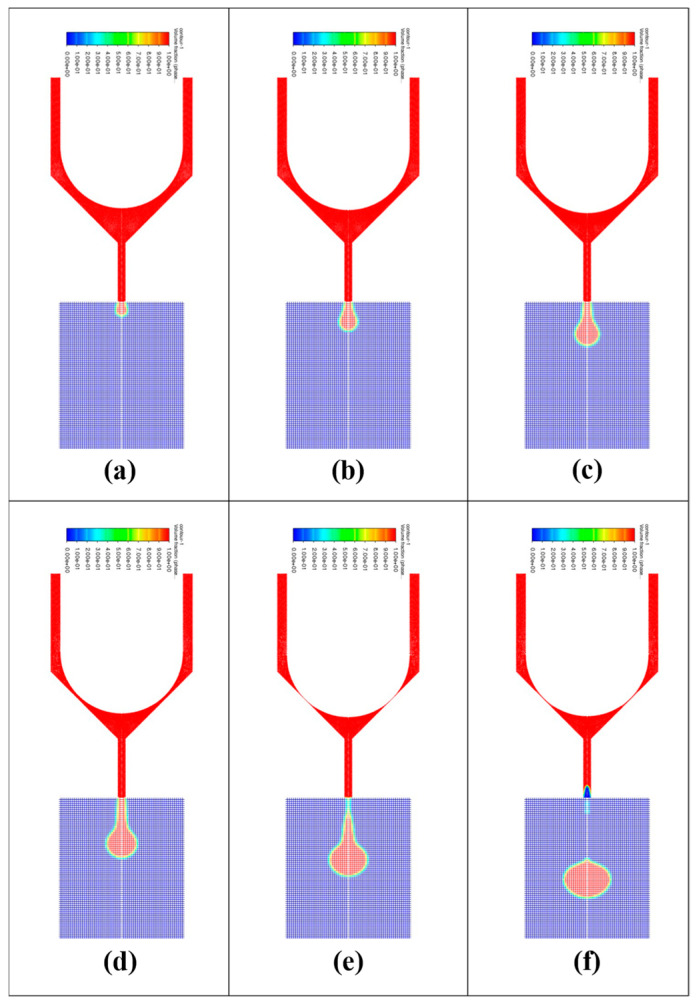
Results of finite element simulation of droplet diameter at different times in case 7: (**a**) time = 0.0002 s, (**b**) time = 0.0004 s, (**c**) time = 0.0006 s, (**d**) time = 0.0008 s, (**e**) time = 0.001 s, (**f**) = 0.0012 s.

Figure 4 and Figure 5 show the results of finite element simulations of velocity variation and droplet diameter during the injection, for case 7. The velocity is measured by placing a virtual probe at point p, and the droplet diameter is measured by calculating the number of meshes occupied by the liquid in the air domain. When the needle moves to the lowest point, the velocity at point p at that moment is defined as the jetting velocity. Figure 4 shows that the velocity at point p increases and then decreases. Figure 5 shows that the droplet diameter first increases, until the droplet is completely detached, and then the droplet diameter remains essentially constant.

**Figure 6:**
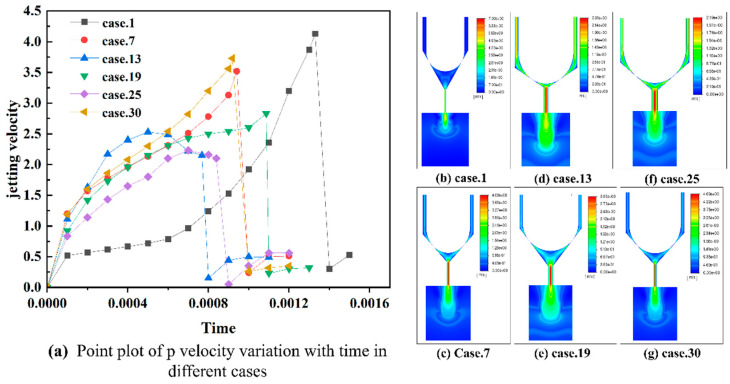
(**a**–**g**) Variation in jetting velocity with time, for different cases.

**Figure 7:**
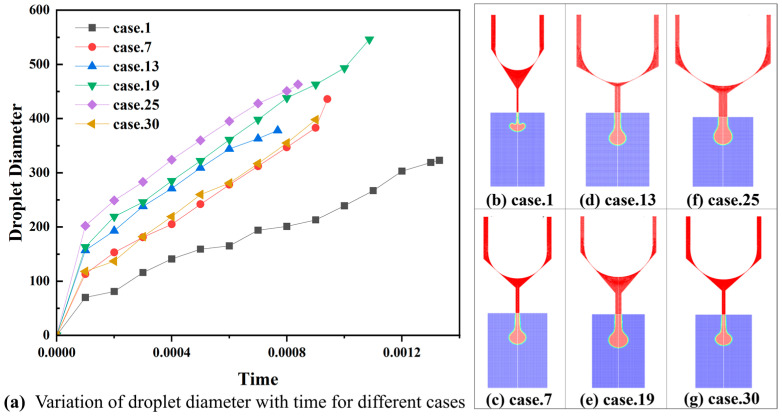
Variation in droplet diameter with time, for different cases. (**a**) variation of droplet diameter with time for different cases, (**b**) case.1, (**c**) case.7, (**d**) case.13, (**e**) case.19, (**f**) case.25, (**g**) case.30.

Figure 6 shows the variation pattern of the jetting velocity for cases 1, 6, 13, 19, 25, and 30, and Figure 7 shows their droplet diameter variation patterns, and also includes the velocity cloud and volume fraction cloud at the time of droplet detachment from the nozzle, indicating that the jetting velocity and droplet diameter variation patterns are different for each case. From the figure, it can be seen that the jetting velocity of case 7 is 3.52 m/s and the droplet diameter at the time of detachment is 436 μm.

## 3. Experimental Verification

**Figure 8:**
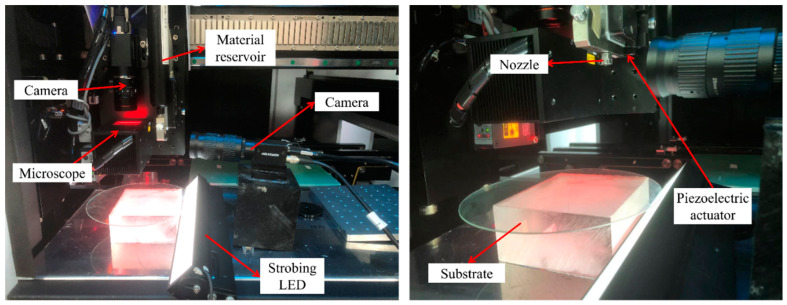
Piezoelectric injection monitoring system.

In this study, a piezoelectric injection monitoring system consisting of a piezoelectric driver, a high-speed camera, an LED, and a host computer was developed, as shown in Figure 8. The image of the piezoelectric injection process is captured by a high-speed camera, and image processing is performed to calculate the displacement of the droplet. The image processing technique can calculate the number of pixels, combine it with the time recorded by the camera, and then obtain the jet speed, according to the displacement time equation. The droplet diameter can also be obtained by acquiring the number of pixels in the area [26,27].

**Figure 9:**
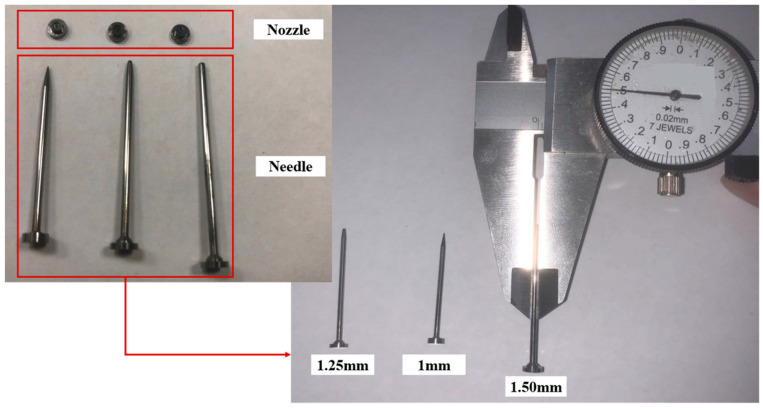
Different sizes of nozzle and needle samples.

**Figure 10:**
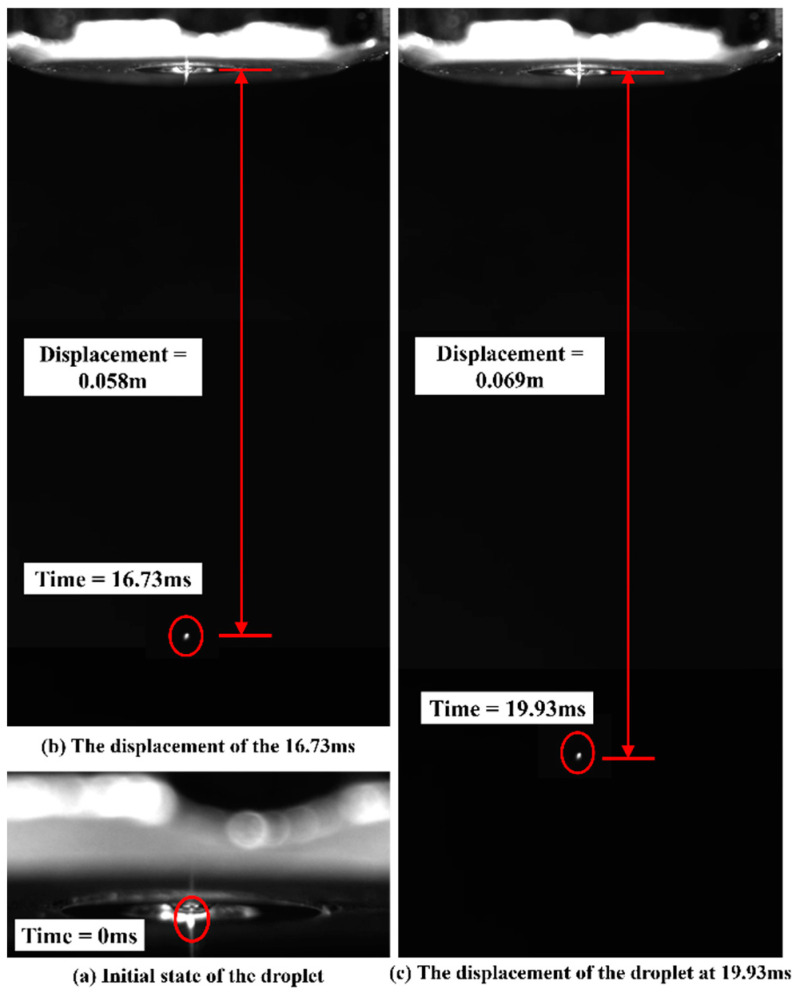
Displacement of the second group of experiments at different moments. (**a**) initial state of the droplet, (**b**) the displacement of the 16.73 ms, (**c**) the displacement of the droplet at 19.93 ms.

**Figure 11:**
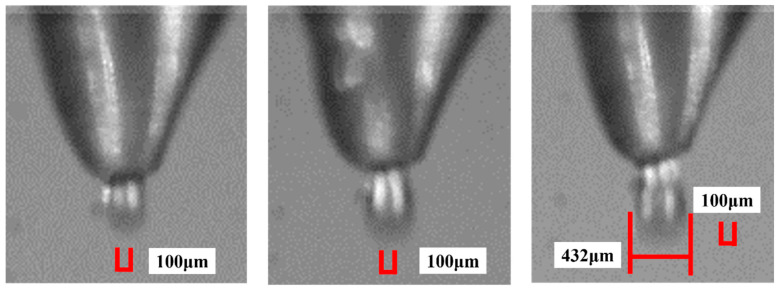
The diameter of droplets at different moments in the second set of experiments.

Case 27 was used as an example for validation. A needle with a diameter of 1.25 mm, and a nozzle with a diameter of 0.16 mm and a taper of 75°, were used in the experiments, and the specific parts are shown in Figure 9. Several measurements and calculations were performed for different injection velocities and droplet diameters. Five sets of experiments were conducted in this study, and each set of experiments recorded data for two different displacements and times, and finally the jet velocity was calculated according to the equation. The specific experimental results are shown in Table 3, and the images captured in the second group of experiments are shown in Figure 10 and Figure 11. To ensure the accuracy of the experimental results, the results of the five sets of experiments were averaged, and the average value of the jetting velocity for case 27 was 3.64 m.s^−1^. Similarly, the average value of the droplet diameter obtained was 463.2 μm.

**Figure 12:**
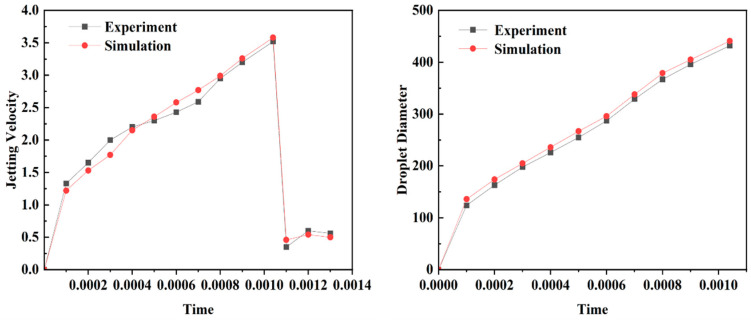
Experimental and simulation comparison of jetting velocity and droplet diameter for case 27.

Figure 12 shows the comparison of the simulation and experimental results. In summary, the jetting velocity predicted by the finite element model established in this paper, is 3.64 m.s^−1^, with an error of 3.02% from the experimental results, and the droplet diameter predicted by the model is 463.2 μm, with an error of 2.20% from the experimental results, which proves that the finite element model proposed in this paper can predict the jetting velocity and droplet diameter accurately.

## 4. Conclusions

This paper presents a method for predicting the performance of piezoelectric injection systems, based on finite element simulations. The obvious advantage of this approach is that it is based on accurate finite element analysis rather than expensive, time-consuming, and error-prone collected experimental data. Coupled modeling of the piezoelectric injection process was performed using the ANSYS Fluent software, and quantitative and qualitative validation of literature data and experiments were performed. Two system performance metrics were proposed: jetting velocity and droplet diameter. A finite element model, with different parameter combinations, was designed, using the Taguchi orthogonal array method. By comparing the predicted and experimental values of the finite element simulation, the error of the jetting velocity was 3.02% and the error of the droplet diameter was 2.20%, indicating that the method proposed in this paper can predict the system performance.

## Figures and Tables

**Table 1:** Fluid material parameters.

Parameter	Fluid Viscosity	Fluid Density	Surface Tension
Value	0.02 Pa.s	1.45 g/cm^3^	0.05 N/m

**Table 2:** Design of parameter combinations based on Taguchi orthogonal method.

Data Case No.	Needle Diameter (mm)	Nozzle Diameter (mm)	Nozzle Taper (°)	Needle Speed m s^−1^	Jetting Velocity m s^−1^	Droplet Diameter (μm)
	N_A_	N_B_	N_C_	N_D_		
1.	1	0.04	60	0.3	4.13	320
2.	1	0.08	75	0.4	4.21	419
3.	1	0.12	90	0.5	3.36	376
4.	1	0.16	105	0.6	2.79	326
5.	1	0.20	120	0.7	2.34	313
6.	1.25	0.04	75	0.7	12.80	247
7.	1.25	0.08	90	0.3	3.52	436
8.	1.25	0.12	105	0.4	2.86	381
9.	1.25	0.16	120	0.5	2.36	338
10.	1.25	0.20	60	0.6	3.78	487
11.	1.5	0.04	90	0.6	16.13	340
12.	1.5	0.08	105	0.7	7.78	317
13.	1.5	0.12	120	0.3	2.13	378
14.	1.5	0.16	60	0.4	3.58	568
15.	1.5	0.20	75	0.5	3.35	476
16.	1.75	0.04	105	0.5	11.00	281
17.	1.75	0.08	120	0.6	6.69	331
18.	1.75	0.12	60	0.7	8.96	520
19.	1.75	0.16	75	0.3	2.85	546
20.	1.75	0.20	90	0.4	2.83	445
21.	2	0.04	120	0.4	10.33	223
22.	2	0.08	60	0.5	11.37	614
23.	2	0.12	75	0.6	8.21	525
24.	2	0.16	90	0.7	6.78	427
25.	2	0.20	105	0.3	2.12	461
26.	1	0.04	60	0.7	15.25	296
27.	1.25	0.16	75	0.5	3.53	453
28.	1.5	0.08	90	0.3	4.08	439
29.	1.75	0.2	105	0.6	3.76	384
30.	2	0.12	120	0.4	3.73	398

**Table 3:** Statistics of the experiment.

Group	Num.	Displacement (m)	Time (s)	Droplet Diameter (μm)	Jetting Velocity (m/s)
Group 1	1	0.055	0.01573	483	3.51
	2	0.066	0.01890		
Group 2	3	0.058	0.01673	432	3.58
	4	0.069	0.01993		
Group 3	5	0.060	0.01699	466	3.69
	6	0.072	0.02042		
Group 4	7	0.073	0.01946	458	3.75
	8	0.085	0.02269		
Group 5	9	0.078	0.02251	477	3.66
	10	0.091	0.02630		
Average value				463.2	3.64

## Data Availability

The data that support the findings of this study are available from the corresponding author upon request.

## References

[1] Murphy S.V., Atala A. (2014). 3D bioprinting of tissues and organs. Nat. Biotechnol..

[2] Yang Y.J., Kim H.C., Sajid M., Kim S.W., Aziz S., Choi Y.S., Choi K.H. (2018). Drop-on-Demand Electrohydrodynamic Printing of High Resolution Conductive Micro Patterns for MEMS Repairing. Int. J. Precis. Eng. Manuf..

[3] Zhang H., Moon S.K., Ngo T.H. (2020). 3D Printed Electronics of Non-contact Ink Writing Techniques: Status and Promise. Int. J. Precis. Eng. Manuf..

[4] Chen F., Zhang Y., Nakagawa Y., Zeng H., Luo C., Nakajima H., Uchiyama K., Lin J. (2013). A piezoelectric drop-on-demand generator for accurate samples in capillary electrophoresis. Talanta.

[5] Salary R.R., Lombardi J.P., Samie Tootooni M., Donovan R., Rao P.K., Borgesen P., Poliks M.D. (2016). Computational Fluid Dynamics Modeling and Online Monitoring of Aerosol Jet Printing Process. J. Manuf. Sci. Eng..

[6] Shin D.Y., Kim M. (2017). Rapid jetting status inspection and accurate droplet volume measurement for a piezo drop-on-demand inkjet print head using a scanning mirror for display applications. Rev. Sci. Instrum..

[7] Mahajan A., Frisbie C.D., Francis L.F. (2013). Optimization of Aerosol Jet Printing for High-Resolution, High-Aspect Ratio Silver Lines. ACS Appl. Mater. Interfaces.

[8] Mao M., He J., Li X., Zhang B., Lei Q., Liu Y., Li D. (2017). The Emerging Frontiers and Applications of High-Resolution 3D Printing. Micromachines.

[9] Bartolo D., Boudaoud A., Narcy G., Bonn D. (2007). Dynamics of Non-Newtonian Droplets. Phys. Rev. Lett..

[10] Mao Z., Asai Y., Yamanoi A., Seki Y., Wiranata A., Minaminosono A. (2022). Fluidic rolling robot using voltage-driven oscillating liquid. Smart Mater. Struct..

[11] Kwon K. (2009). Speed measurement of ink droplet by using edge detection techniques. Measurement.

[12] Mao Z., Asai Y., Wiranata A., Kong D., Man J. (2022). Eccentric actuator driven by stacked electrohydrodynamic pumps. J. Zhejiang Univ. Sci. A.

[13] Tsai M.H., Hwang W.S., Chou H.H., Hsieh P.H. (2008). Effects of pulse voltage on inkjet printing of a silver nanopowder suspension. Nanotechnology.

[14] Swaminathan C.R., Voller V.R. (1994). A time-implicit filling algorithm. Appl. Math. Model..

[15] Pan Y., Zeng L. (2019). Simulation and Validation of Droplet Generation Process for Revealing Three Design Constraints in Electrohydrodynamic Jet Printing. Micromachines.

[16] Zhong Y., Fang H., Ma Q., Dong X. (2018). Analysis of droplet stability after ejection from an inkjet nozzle. J. Fluid Mech..

[17] Liou T., Chan C., Shih K. (2010). Effects of actuating waveform, ink property, and nozzle size on piezoelectrically driven inkjet droplets. Microfluid. Nanofluid..

[18] Liu Y., Derby B. (2019). Experimental study of the parameters for stable drop-on-demand inkjet performance. Phys. Fluids.

[19] Ktari A., El Mansori M. (2021). Intelligent approach based on FEM simulations and soft computing techniques for filling system design optimisation in sand casting processes. Int. J. Adv. Manuf. Technol..

[20] Feng Y., Liu J., Li K., Li H., Deng J., Liu Y. (2022). Waveform Optimization of Piezoelectric Micro-Jet for the Control of Metal Micro-Droplet Ejection. IEEE Trans. Ind. Electron..

[21] Huang J., Segura L.J., Wang T., Zhao G., Sun H., Zhou C. (2020). Unsupervised learning for the droplet evolution prediction and process dynamics understanding in inkjet printing. Addit. Manuf..

[22] Falahat A. (2023). Sensitivity analysis of boehmite alumina nanofluid in a novel cylindrical heat sink with hybrid helical-straight minichannels using the taguchi method and statistical analysis. Int. J. Therm. Sci..

[23] Vidakis N., Petousis M., Mountakis N., Moutsopoulou A., Karapidakis E. (2023). Energy Consumption vs. Tensile Strength of Poly[methyl methacrylate] in Material Extrusion 3D Printing: The Impact of Six Control Settings. Polymers.

[24] Aramli M.S., Sarvi Moghanlou K., Imani A. (2023). Effect of dietary antioxidant supplements (selenium forms, alpha-tocopherol, and coenzyme Q10) on growth performance, immunity, and physiological responses in rainbow trout (Oncorhynchus mykiss) using orthogonal array design. Fish Shellfish. Immunol..

[25] Shirpurkar P.P., Kamble P.D., Bobde S.R., Patil V.V. (2014). Optimization of CNC Turning Process Parameters for Prediction of Surface Roughness by Taguchi Orthogonal Array.

[26] Ball A.K., Das R., Das D., Roy S.S., Murmu N.C. (2018). Design, Development and Experimental Investigation of E-jet Based Additive Manufacturing Process. Mater. Today Proc..

[27] De Ferreira B.C., Coutinho T., Ayala Botto M., Cardoso S. (2022). Development of an Inkjet Setup for Printing and Monitoring Microdroplets. Micromachines.

